# Let Others Buy First: Identity Fusion Buffers the Effect of COVID-19 Phobia on Panic Buying Behavior From an Economic Perspective

**DOI:** 10.3389/fpsyg.2021.710185

**Published:** 2021-09-27

**Authors:** Yi-Tai Seih, Vi Thanh Tra, Marketa Lepicovsky, Yi-Ying Chang

**Affiliations:** Department of Business Administration, National Taiwan University of Science and Technology, Taipei City, Taiwan

**Keywords:** identity fusion, COVID-19, coronavirus, phobia, panic buying behavior

## Abstract

The coronavirus 2019 (COVID-19) pandemic has caused hundreds of millions of cases and millions of deaths, resulting in the development of COVID-19 phobia. To prevent getting COVID-19, Centers for Disease Control and Prevention (CDC) in many countries encourage people to protect themselves via several strategies, such as wearing face masks or using sanitizers when washing hands. However, at times, such supplies for preventing COVID-19 are limited. In this study, we examine the relationship between COVID-19 phobia and panic buying behavior from an economic perspective and test if identity fusion plays a buffering role for this phenomenon. Data was collected from September 4th to November 1st in 2020 across three countries (the United States, Germany, and Taiwan). A self-report measure of panic buying behavior was developed and culturally cross-validated. Moderation analyses were conducted focusing on the study objectives. Results show that the economic factor in COVID-19 phobia predicts panic buying behavior, and this effect is buffered by identity fusion. It is worthy to note that this buffering effect emerged only in the Taiwanese sample, not in the American or German samples. Implications of identity fusion theory in human behavior are discussed.

## Introduction

The outbreak of the COVID-19 pandemic thrust a new worry into the everyday consciousness of people around the world. Within a matter of weeks, the attention of governments and citizens in many countries shifts to the deadly new virus and how to maintain health and safety in communities. This situation is a source of fear and anxiety for many. With the onset of the pandemic, researchers began to investigate COVID-19 related fear and anxiety (e.g., Lee et al., 2020) and thereby shed light on how individuals psychologically perceive and behaviorally react to COVID-19 during the ongoing pandemic. For example, people worry about the impact of COVID-19 on their overall health (Jungmann and Witthöft, 2020), and such psychological difficulties might lead to a number of negative mental health outcomes (Lee et al., 2020).

The COVID-19 pandemic has serious adverse effects on the global economy. Government led pandemic response efforts have disrupted the supply chain in numerous industry sectors (Del Río-Chanona et al., 2020). Many companies have been ordered to shut down their businesses to prevent the spread of the virus (Billore and Anisimova, 2021), causing a vast increase in unemployment, declines in production, and shortages of necessary goods and supplies. For example, personal protective equipment was reported to be in limited supply during the crisis (Burki, 2020). These supply disruptions may intensify people’s anxiety about a potential shortage or increase in the price of goods. Alongside health-related concerns, concerns related to the economic consequences of COVID-19 are a likely source of psychological distress (Mann et al., 2020). In this context, the current research sought to examine how these psychological issues are related to panic buying by adopting an economic point of view.

In order to understand how different components, including economic challenges, contribute toward psychological distress, Arpaci et al. (2020) developed a scale to measure COVID-19 phobia, which refers to an overwhelming and debilitating fear or anxiety about COVID-19. This novel COVID-19 phobia construct is comprised of four distinct factors, including psychological, psycho-somatic, social, and economic factors (Arpaci et al., 2020). The psychological factor involves a feeling of fear and uncertainty about the disease and its health-related consequences. This means, people are afraid of being ill or dying from a specific disease. The psycho-somatic factor refers to physiological problems caused by anxiety, such as stomachaches or sleep problems (Lee, 2020). The social factor suggests a social distance people are willing to maintain with others (Olivera-La Rosa et al., 2020) because of the contagious nature of COVID-19. This fear of COVID-19 might influence interpersonal social interactions between people. Last, during times of crisis, fear and the worry of being inadequately prepared leads people to stock up on food and supplies (Larson and Shin, 2018). Thus, anxiety related to shortages of food or medical supplies, such as face masks, reflects a COVID-19 phobia of an economic nature. This phobia may lead people to engage in extreme or unusual behaviors, such as panic buying.

Panic buying behavior is defined as a goal-directed or impulsive behavior (Arafat et al., 2020c; Taylor, 2021), which involves a sudden purchase of a large volume or a variety of products in excess of regular need. Arafat et al. (2020c) posit that panic buying is often observed during a time of crisis, disruptive events, or ceremonial occasions and could result in a supply-demand imbalance. Such behavior may occur under the influence of negative mood states or feelings (e.g., Lee et al., 2020). When confronted with an uncontrollable crisis, people experience stronger feelings of anxiety, fear, or uncertainty about the future. In these circumstances, panic buying behavior is used to cope with these negative mood states and regain a sense of control over the situation (Yuen et al., 2020).

Previous research has explored psychological explanations for panic buying during a time of crisis (Arafat et al., 2020a,b, 2021). Several factors have been discussed, including the perception of scarcity, a sense of losing control or security, social learning, lack of trust, government policies, and past experience. In a content analysis of 613 media reports by Arafat et al. (2020b), some of the most frequently identified reasons behind panic buying include the perception of scarcity and an increased demand for commodities. This implies the psychological concerns related to panic buying are primarily economic in nature.

Panic buying behavior has been observed at various times during the COVID-19 pandemic. In order to stop the spread of COVID-19 through interpersonal interactions, some countries impose lockdown orders and flight restrictions. These governmental policies for controlling COVID-19 may induce panic buying. For example, travel bans contribute to economic recession in the airline industry. The flight restrictions further cause a desperate shortage of essential commodities, such as face masks, that sparks panic buying behavior. In addition, stay-at-home orders implemented by many countries not only sharply reduces travel but also increases the need for daily and medical supplies. The need to maintain enough food and supplies to enable staying home for weeks emerges as a substantial concern. This may result in shortages of food and supplies, and the fear may trigger widespread panic buying (Li et al., 2020). It is believed to be a psychologically driven consumption phenomenon linked to psychological distress (Yuen et al., 2020).

If panic buying behavior is psychologically motivated by individuals’ anxiety or fear, which are indicative of a phobia, could COVID-19 related phobia predict this unusual behavior? Researchers investigated COVID-19 from a phobia perspective (Arpaci et al., 2020). They specifically defined a new concept, COVID-19 phobia and identified four factors in the construct of COVID-19 phobia. Among the four factors, the economic factor is conceptually related to panic buying behavior the most closely. Items in the economic factor in the COVID-19 phobia scale describe situations concerning supply shortages and stockpiling. From an economic point of view, if people have concerns about supply shortages and start to stockpile because of the economic factor in COVID-19 phobia, this may induce their motivation to engage in more panic buying behavior.

Previous COVID-19 research suggests pandemic panic buying is driven by the economic factor in COVID-19 phobia including worries about shortages (Kaur and Malik, 2020) and fears of associated price increases (Arafat et al., 2020c). First, people may amass excessive amounts of supplies to avoid feeling unprepared. This behavior could increase in a crisis situation, as people become more risk-averse. During the pandemic, people stockpile due to a feeling of not having enough food or supplies (Dammeyer, 2020).

Second, when people perceive problems with the availability of certain products, their need for the threatened items increases (Clee and Wicklund, 1980). People may become nervous when they sense that the availability of products or time for shopping are scarce and begin to buy insanely as a result. For example, research indicates that during the pandemic, people are willing to pay more for fresh food and buy it in excessive amounts (Wang E. et al., 2020). Based on findings about COVID-19 and panic buying, we hypothesize that the economic factor in COVID-19 phobia could predict panic buying behavior (Hypothesis 1).

Panic buying may cause deviant behavior or even social chaos. Rumors about the shortages of daily or medical supplies are spread rapidly on public and social media, leading people to line up outside supermarkets and pharmacies. Although government officials call on people to remain calm, the panic situation is not controlled very well. Given that people who suffer from COVID-19 phobia may display panic buying, is there any approach that could decrease the occurrence of panic buying behavior? To our knowledge, the construct of identity fusion may be a key to answer this question.

The identity fusion theory states that the more closely individuals align their personal identity with that of a group, the more likely they are to engage in and endorse pro-group behaviors that bear a personal cost (Swann et al., 2009). In other words, people who are strongly fused with a group are more likely to self-sacrifice for the good of the group (Chaudhuri, 2011). For instance, individuals whose identity is more fused with their country are more willing to fight and die for their nation (Swann et al., 2010a). Gómez. (2020) argues that identity fusion could lead people to take up greater self-sacrificing behavior in the context of the COVID-19 pandemic. This suggests that identify fusion may affect individual panic buying behavior in a pandemic context.

Past research about identity fusion found that in a crisis or disaster situation, people who are fused with group members contribute toward helping behavior (Drury et al., 2009). A recent study found that individuals with high identity fusion are more likely to let others get vaccinated first when facing a deadly virus than in the less serious condition of an illness causing virus (Paredes et al., 2020). This finding implies that identity fusion could play a buffering role when people display panic behavior caused by COVID-19 phobia. During the pandemic, people may experience fear or anxiety about shortages of food or medical supplies due to COVID-19 phobia, particularly the economic factor in COVID-19 phobia. However, according to previous findings on identity fusion, individuals who are highly fused with their identity group may let others buy food or medical supplies first, thereby sacrificing themselves to face the threat of COVID-19. Thus, we hypothesize that identity fusion could function as a buffer to moderate the effect of the economic factor in COVID-19 phobia on panic buying behavior (Hypothesis 2).

## Materials and Methods

### Participants and Procedures

Data were obtained from September 4th to November 1st in 2020 and collected from three countries. At the time of the study, confirmed cases of COVID-19 mainly occurred in countries located in North America, Europe, and Asia, according to the World Health Organization’s (WHO) dataset.^1^ Therefore, we selected one country from each represented continent. The inclusion criterion we set is the country has experienced an outbreak, epidemic, or pandemic after the year 2000. The United States experienced swine flu (H1N1) in 2009 and influenza A H3N2 variant virus from 2017 to 2018. In 2009, Germany was heavily impacted by a swine flu epidemic. Taiwan experienced a series of severe acute respiratory syndrome coronavirus (SARS) outbreaks in 2003. The United States, Germany, and Taiwan have all experienced an epidemic or pandemic in the recent years prior to COVID-19. Based on this common past experience, the present study is conducted in the context of these three countries.

In the American sample, 227 participants (132 females) were recruited from Amazon’s Mechanical Turk (MTurk). MTurk is commonly used for academic purposes and has become a popular online data collection platform (Buhrmester et al., 2011). The age of the American participants ranges from 18 to 70 (*M*_*age*_ = 36.75, *SD* = 11.14). All participants are native English speakers from the United States, and 96% of them are American citizens. The ethnic composition is 78.0% White, 13.2% African American, 5.3% Asian American, 2.6% Hispanic, and 0.9% other. Participants were paid 25 cents for completing our study on MTurk.

In the German sample, 247 participants (118 females) were recruited from several popular social network sites (SNSs), including Facebook, Instagram, and Twitter. The age of the German participants ranges from 18 to 75 (*M*_*age*_ = 30.24, *SD* = 8.09). The nationality composition was 94.9% German and 5.1% other. All participants are native German speakers, and they voluntarily participated in this study. We thanked them for their participation after they finished the survey.

In the Taiwanese sample, two hundred and sixty participants (162 females) were recruited from several popular SNSs, including Facebook, Instagram, and a popular Taiwanese bulletin board system (PTT). The age of the Taiwanese participants ranges from 19 to 72 (*M*_*age*_ = 38.64, *SD* = 13.09). All participants are native Chinese speakers and Taiwanese citizens. They voluntarily participated in this study, and we thanked them for their participation after they finished the survey. A detailed overview of the demographic information is presented in Table 1.

**TABLE 1:** Descriptive statistics of the participants in the present study.

**Sample**		**American (*N* = 227)**	**German (*N* = 247)**	**Taiwanese (*N* = 260)**	**Total (*N* = 734)**	
					**Frequency**	**%**
Gender	Male	95	129	98	322	43.9%
	Female	132	118	162	412	56.1%
Age	18–29	83	155	93	331	45.1%
	30–39	53	70	31	154	21.0%
	40–49	68	17	64	149	20.3%
	50–59	18	2	55	75	10.2%
	Above 60	5	3	17	25	3.4%
Education	High School	15	18	31	64	8.8%
	Completed Apprenticeship	18	105	52	175	23.8%
	Bachelor’s Degree	129	77	103	309	42.1%
	Master’s Degree or Higher	65	47	74	186	25.3%
Work status	Student	19	117	39	175	23.8%
	Employed Full-Time	176	87	164	427	58.2%
	Employed Part-Time	14	22	27	63	8.6%
	Self-Employed	15	8	11	34	4.6%
	Employment Seeker	1	6	2	9	1.3%
	Other	2	7	17	26	3.5%

### Measures

All measurement tools in this study were developed in English; therefore, the English version of all scales were used for the American sample. For the German sample, English scales were translated into the German language by using the back translation method (Geisinger, 1994). The back translation method was also applied for translating English scales into the Chinese language for the Taiwanese sample.

*COVID-19 phobia*: COVID-19 phobia was measured through the COVID-19 Phobia Scale (C19P-S) developed by Arpaci et al. (2020). The C19P-S contains four factors with 20 items in a 5-point scale and was used to assess to what degree people feel afraid of or anxious about COVID-19. The four factors include psychological, psycho-somatic, economic, and social factors. A sample item from the economic factor in COVID-19 phobia factor is “*The possibility of food supply shortage due to the coronavirus pandemic causes me anxiety.*” The Cronbach’s alphas are 0.86 for the psychological factor, 0.95 for the psycho-somatic factor, 0.89 for the economic factor, and 0.81 for the social factor in this study.

*Identity fusion:* The identity fusion scale was developed by Gómez et al. (2011). This scale contains seven items in a 5-point scale to assess feelings of reciprocal strength between individuals and a group. In this study, the group in the identity fusion scale refers to home country. A sample item is “*I feel immersed in my country.*” The Cronbach’s alpha is 0.91 in this study.

*Panic buying behavior:* We created five items to tap into participants’ panic buying behavior, such as stocking up on daily/medical supplies or getting in line for daily/medical supplies (see Appendix). The five items are a dichotomous scale, and participants responded to the items with either yes (1) or no (0). The sum score ranges from 0 to 5. Higher scores reflect greater panic buying behavior during the COVID-19 pandemic. A sample item is “*During the COVID-19 outbreak, at least once, I have flocked to brick-and-mortar/online stores for stocking up on medical supplies, such as face masks, sanitizers, etc.*” The Cronbach’s alpha is 0.75 in this study.

## Results

### Face Validity

Because the five items measuring panic buying behavior were created by the researchers of this study, face validity tests were conducted in each of the three countries respectively. Five social psychologists in the US (2 females) were invited to rate the English version, four social psychologists in Germany (2 females) were invited to rate the German version, and four social psychologists in Taiwan (1 female) were invited to rate the Chinese version. The question, “This item appears to measure what it is supposed to mean,” answered according to a 5-point scale ranging from 1 (strongly disagree) to 5 (strongly agree) was used to evaluate face validity. Across the 5 items, the average rating scores were 4.68 (*SD* = 0.18) from the American psychologists, 4.70 (*SD* = 0.20) from the German psychologists, and 4.70 (*SD* = 0.12) from Taiwanese psychologists. If we merged the rating scores from the 13 social psychologists, the final average rating score was 4.69 (*SD* = 0.16). Items rated higher than 4 on average were considered to have adequate expert validity. All of the 5 items reached this criterion.

### Descriptive Statistics and Correlations

Descriptive statistics and correlations across the three countries are provided in Table 2. Before testing our hypotheses, we examined relationships between variables. In the table, the independent variable is the economic factor in COVID-19 phobia, while the dependent variable is panic buying behavior, and the moderator is identity fusion. Other variables are considered supplementary variables. Results showed that gender is negatively related to psychological (*r* = −0.10, *p* < 0.05) and social (*r* = −0.07, *p* < 0.05) COVID-19 phobia, suggesting that females reported higher scores on psychological and social COVID-19 phobia compared to males. There is no gender difference on identity fusion (*r* = −0.02, *p* = 0.58) and panic buying behavior (*r* = −0.04, *p* = 0.24). In terms of age, it is positively related to psychological (*r* = 0.12, *p* < 0.01), psycho-somatic (*r* = 0.18, *p* < 0.01), economic (*r* = 0.22, *p* < 0.01), and social (*r* = 0.23, *p* < 0.01) COVID-19 phobia, indicating that older people have stronger feelings of fear and anxiety related to COVID-19 and reported higher scores on the four factors reflecting COVID-19 phobia. Furthermore, age is positively related to identity fusion (*r* = 0.14, *p* < 0.01) and panic buying behavior (*r* = 0.13, *p* < 0.01). This indicates that older people are more fused with their country identity and also reported more panic buying behavior during the COVID-19 pandemic.

**TABLE 2:** Descriptive statistics and correlations across the three countries (*N* = 734).

**Variable**	**Mean (*SD*)**	**1**	**2**	**3a**	**3b**	**3c**	**3d**	**4**	**5**
1. Gender	0.49(0.50)								
2. Age	34.89(12.06)	0.08*							
3. COVID-19 Phobia									
3a. Psychological	3.09(0.88)	−0.10**	0.12**	(0.86)					
3b. Psycho-somatic	2.19(1.20)	0.03	0.18**	0.72**	(0.96)				
3c. Social	3.21(0.90)	−0.07*	0.23**	0.82**	0.69**	(0.81)			
3d. Economic	2.64(1.11)	–0.03	0.22**	0.75**	0.84**	0.77**	(0.89)		
4. Identity fusion	3.83(1.18)	–0.02	0.14**	0.06	0.03	0.11**	0.14**	(0.91)	
5. Panic buying behavior	2.34(1.05)	–0.04	0.13**	0.49**	0.63**	0.61**	0.51**	0.07*	(0.78)

Gender: 1 = Male, 0 = Female. Cronbach’s alphas are provided in brackets along the diagonal. *p < 0.05, **p < 0.01 (two-tailed).

Next, we examined country differences on our variables by conducting one-way ANOVA tests. Results are shown in Table 3. Across the four factors reflecting COVID-19 phobia, Scheffe *post hoc* tests for pairwise comparisons indicate that participants in the American sample have higher scores than those in the German and Taiwanese samples; participants in the Taiwanese sample have higher scores than those in the German sample. These findings are consistent across the four COVID-19 phobia factors between the three samples. Similar findings also emerged in panic buying behavior. Participants in the American samples reported more panic buying behavior than those in the German and Taiwanese samples; participants in the Taiwanese sample reported more panic buying behavior than those in the German sample. However, this pattern changed in identity fusion. Participants in the Taiwanese sample reported higher scores than those in the American and German samples; participants in the American sample reported higher scores than those in the German sample.

**TABLE 3:** One-way ANOVA tests on each of the study variables across the three countries.

	**United States (*N* = 227)**	**Germany (*N* = 247)**	**Taiwan (*N* = 260)**		

	**Mean (*SD*)**			***F***	**η *2_p_***
COVID-19 Phobia					
Psychological	3.78 (0.70)^*a*^	2.55 (0.78)^*b*^	3.01 (0.69)^*c*^	171.50***	0.32
Psycho-somatic	3.63 (0.86)^*a*^	1.23 (0.41)^*b*^	1.83 (0.68)^*c*^	819.71***	0.69
Social	3.85 (0.70)^*a*^	2.47 (0.74)^*b*^	3.36 (0.67)^*c*^	235.93***	0.39
Economic	3.74 (0.72)^*a*^	1.57 (0.59)^*b*^	2.68 (0.72)^*c*^	600.61***	0.62
Identity fusion	3.79 (1.32)^*a*^	3.53 (1.07)^*b*^	4.15 (1.05)^*c*^	18.41***	0.05
Panic buying behavior	3.70 (1.30)^*a*^	1.42 (1.28)^*b*^	2.03 (1.46)^*c*^	179.16***	0.33

^***^p < 0.001. ^abc^Means in a row without a common superscript letter differ (p < 0.05) as analyzed by one-way ANOVA and the Tukey HSD tests.

### Hypothesis Tests

After conducting correlation and ANOVA tests, we examined the two hypotheses. In our regression analysis, gender and age were entered in step 1 as control variables, and the economic factor in COVID-19 phobia was entered in step 2. Results showed that the regression model 1 was significant, [*F*(2, 731) = 7.94, *p* < 0.001], with an adjusted *R*^2^ of.02. In the regression model 2 [*F*(3, 730) = 114.58, *p* < 0.001], the economic factor in COVID-19 phobia (β = 0.90, *SE* = 0.04, *p* < 0.001) significantly predicted panic buying behavior, with a significant adjusted *R*^2^ change of.35 (*p* < 0.001). Our findings indicate that the economic factor in COVID-19 phobia could positively predict panic buying behavior, supporting our H1. To ensure this finding is stable across the three countries, we broke down data by countries and ran the same regression analysis. All findings are the same, and this confirms our findings are stable across countries.

**FIGURE 1:**
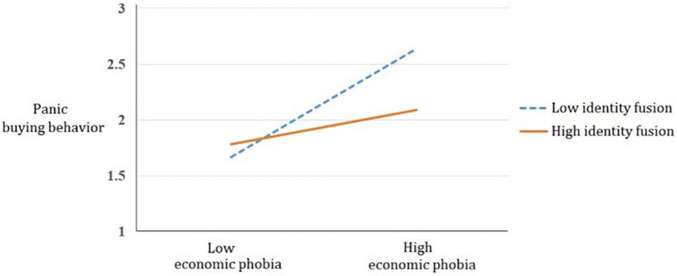
The two-way interaction effect of the economic factor in COVID-19 phobia and identity fusion on panic buying behavior in the Taiwanese sample.

To test H2, moderation analysis in the SPSS PROCESS macro (model 1) with 5,000 bootstrapping (Hayes, 2017) was used. The predictor is the economic factor in COVID-19 phobia, the moderator is identity fusion, control variables are gender and age, and the outcome variable is panic buying behavior. The results showed no significant interaction effect (β = −0.05, *SE* = 0.04, *p* = 0.15, *LLCI* = −0.12, *ULCI* = 0.02) with the three samples. Therefore, we examined this moderation effect within each sample. There is no significant interaction effect in the American sample (β = −0.07, *SE* = 0.06, *p* = 0.31, *LLCI* = −0.22, *ULCI* = 0.07) nor in the German sample (β = 0.04, *SE* = 0.11, *p* = 0.68, *LLCI* = −0.17, *ULCI* = 0.26). However, the interaction effect between the economic factor in COVID-19 phobia and identity fusion on panic buying behavior is significant in the Taiwanese sample (β = −0.23, *SE* = 0.10, *p* = 0.03, *LLCI* = −0.44, *ULCI* = −0.02). To interpret this effect, we plotted the interaction between the economic factor in COVID-19 phobia and identity fusion based on the recommendations of Cohen et al. (2003) (see Figure 1). The corresponding simple effects were calculated by SPSS PROCESS macro (Hayes, 2017).

The results showed that among Taiwanese participants with high the economic factor in COVID-19 phobia (*Mean* + 1*SD*), those with higher identity fusion display less panic buying behavior, compared to those who have lower identity fusion (β = −0.31, *SE* = 0.11, *p* < 0.01, *LLCI* = −0.54, *ULCI* = −0.09). Nevertheless, Taiwanese participants with low the economic factor in COVID-19 phobia (*Mean*—1*SD*) display the same level of panic buying behavior (β = 0.03, *SE* = 0.10, *p* = 0.82, *LLCI* = −0.20, *ULCI* = 0.25) whether they have higher or lower identity fusion.

## Discussion

The present research investigates if identity fusion could moderate the effect of the economic factor in COVID-19 phobia on panic buying behavior. First, the correlational results showed that as the economic factor in COVID-19 phobia increased, so did panic buying. This indicates that the economic factor in COVID-19 phobia might induce panic buying behavior. The results from one-way ANOVA tests suggest that American people might suffer more from COVID-19 phobia and engage more in panic buying, compared to other countries.

It is worth to note that Taiwanese participants reported higher scores than American and German participants on identity fusion, suggesting that Taiwanese people are more likely to commit self-sacrificing behavior when facing the threat of the COVID-19 pandemic. Drawing on the identity fusion theory, the current study tested identity fusion as a moderator. Across the three countries, the moderation effect did not emerge. However, when we tested the moderation effect within each country, the effect only emerged in the Taiwanese sample. This demonstrates that Taiwanese people, who perceived higher the economic factor in COVID-19 phobia and reported a stronger level of identity fusion, display fewer occurrences of panic buying behavior.

### Theoretical Contributions

The present research provides ecological evidence to extend the identity fusion theory. The majority of studies on identity fusion instruct participants to imagine scenarios and to perform hypothetical behavior (e.g., Swann et al., 2010a). Such decisions from imaginary situations could not reflect real actions in social contexts (FeldmanHall et al., 2012). Collecting behavioral responses to the COVID-19 pandemic furthers the understanding of how identity fusion functions in the context of dynamic, real-world events rather than in laboratory settings.

The main findings from the current research illustrates that identity fusion could serve as a moderator of actual human behavior. Past literature related to the identity fusion theory focuses on the willingness to self-sacrifice for the group (e.g., Swann et al., 2010b), but Sheeran and Webb (2016) indicated a research gap between intentions and actual behaviors, in which people are unlikely to translate their intentions to real actions. Our findings help fill this research gap and confirm the identity fusion could help account for actual behaviors, especially in an extreme situation. More importantly, our results support recent findings about identity fusion on the COVID-19 pandemic, such as self-sacrificing behavior (Paredes et al., 2020). Additionally, past studies have mostly focused on samples from western countries (e.g., Newson et al., 2016; Swann et al., 2010a,b). Our study contributes to the development of the identity fusion theory by extending its application to different cultures.

### Practical Implications

The result shows that Taiwanese people, who are highly fused to their country, display self-sacrificing behavior reflected in reduced panic buying. They pass up the opportunity to stock up on supplies for themselves and thus give priority for personal protective equipment (e.g., masks) to healthcare workers. A possible explanation is that positive group experiences or events could foster identity fusion (Gómez, 2020). This positive group experience might be attributed to the effective COVID-19 prevention policies that the Taiwanese government implemented nationwide. For instance, to preventing stockpiling, a mask-rationing plan guaranteed people have access to equal amounts of masks at contracted pharmacies. Further, all passengers on public transportation systems throughout Taiwan are required to wear masks. These policies have been implemented nationwxside in Taiwan, and in this respect, they differ from regional policies such as lockdowns. Regional policies only affect people in specific areas, but nationwide policies instill a sense among people that their government is fighting against COVID-19 at the national level.

The nationwide policies stabilized the situation in Taiwan during the outbreak of the COVID-19 pandemic. After experiencing severe acute respiratory syndrome (SARS) in 2003, Taiwan remains vigilant when facing COVID-19, leading the government to implement proactive policies in response to the outbreak (Capano et al., 2020). Effective policies might make people feel protective, and this positive experience seems to be the potential factor that influences identity fusion found in the Taiwanese sample. This result is in line with the correlational evidence between euphoric event and identity fusion (e.g., Newson et al., 2016). Furthermore, the size of countries might be an alternative explanation. The present research investigates three countries, and Taiwan is a small island country compared to other two countries. It is relatively more difficult to implement nationwide policies in continental countries because of greater landmass. In larger geographical areas, implementing governmental policies effectively against COVID-19 becomes a challenge.

### Limitations and Future Directions

Our main findings are observed in the Taiwanese sample. First, from a generalization perspective, Taiwan cannot represent all countries in Asia due to the different implemented policies across different countries. Future research could extend the research to other countries in Asia, and a cross-national study in Asia would provide more interesting and fruitful findings for further research. Second, identity fusion is measured by a self-report scale in our study. Future research could cross-validate our findings with other methods, such as experimental design to manipulate identity fusion. Third, the validity of the instrument to measure panic buying behavior is assessed by face validity. Future researchers are encouraged to perform different validity tests (e.g., convergent or discriminant validity) to cross-validate this measurement.

Finally, in our findings, the moderation effect was found only in Taiwan, and not in the other two countries. Hence, implemented policies might play an important role in managing panic buying during the COVID-19 pandemic. Moreover, it is possible that additional factors contribute to this inter-country difference. For example, trust in authority could be a potential moderator that may mitigate panic buying. Arafat et al. (2020a) argue that lack of trust might lead to increased fear among citizens and thereby contributes toward panic buying. Huang (2020) also supports this viewpoint, stating that the Taiwanese have high trust in the government, thus they are more willing to support each other’s efforts in keeping their community healthy. In comparison to Taiwan, the other two countries may experience less confidence in their governments. This speculation was reflected by the score of identity fusion presented in the current study, which is also an indicator of intragroup trust.

In addition, communication patterns could contribute to the explanation of this moderating effect. People may feel threatened and begin to panic due to ineffective communication by the government or the media (Arafat et al., 2020a). For example, media reports may increase fear and worry in the community by displaying visual images of empty supermarket shelves or long lines outside the stores. Such information presented in the media might provoke people to engage in panic buying. Compared to other countries, the Taiwanese government conducts daily press conferences, answering questions raised by reporters and making announcements regarding the danger of stockpiling (Wang C.J. et al., 2020). Such effective communication could make people learn that the government is committed to preventing the spread of the corona virus. People might feel protected and tend to engage less in harmful social behaviors. Future research could investigate how governmental policies regarding COVID-19 and these proposed factors influence panic buying behavior.

## Conclusion

The present research provides insights into the understanding of panic buying patterns, suggesting that people might engage in panic buying behavior when perceiving the economic factor in COVID-19 phobia. Governments could implement effective policies to make people feel fused with their country for reducing panic buying behavior. Identity fusion can be a buffer when people feel anxious and engage in panic buying. Thus, we hope policymakers in government will understand the importance of identity fusion in promoting prosocial behaviors when their country faces extreme situations or crises.

## Data Availability Statement

The raw data supporting the conclusions of this article will be made available by the authors, without undue reservation.

## Ethics Statement

The studies involving human participants were reviewed and approved by Institutional Review Board, National Taiwan University. The patients/participants provided their written informed consent to participate in this study.

## Author Contributions

Y-TS and VTT contributed to the study design and collected, analyzed, and interpreted the data. Y-TS, VTT, and ML wrote the manuscript. Y-YC provided the critical feedback. All authors contributed to the article and approved the submitted version.

## Conflict of Interest

The authors declare that the research was conducted in the absence of any commercial or financial relationships that could be construed as a potential conflict of interest.

## Publisher’s Note

All claims expressed in this article are solely those of the authors and do not necessarily represent those of their affiliated organizations, or those of the publisher, the editors and the reviewers. Any product that may be evaluated in this article, or claim that may be made by its manufacturer, is not guaranteed or endorsed by the publisher.

## Footnotes

1
https://www.who.int/data

## Appendix

### Panic Buying Behavior Items

According to your own behavior, please select the reply that best describes yourself.

**Table:**

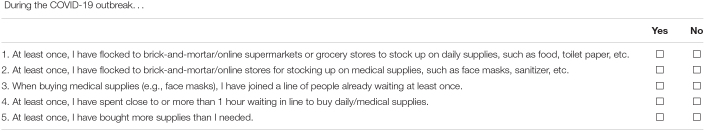

### Scoring of the Panic Buying Behavior Items

The five panic buying behavior items form a self-report, dichotomous scale to assess the individual levels of panic buying behavior. All items are responded to with either “yes (1)” or “no (0).” The scores range between 0 to 5. A higher score indicates individuals display more panic buying behavior.
